# Mechanisms of PKA-Dependent Potentiation of Kv7.5 Channel Activity in Human Airway Smooth Muscle Cells

**DOI:** 10.3390/ijms19082223

**Published:** 2018-07-30

**Authors:** Lyubov I. Brueggemann, Leanne L. Cribbs, Jeffrey Schwartz, Minhua Wang, Ahmed Kouta, Kenneth L. Byron

**Affiliations:** 1 Department of Molecular Pharmacology & Therapeutics, Loyola University Chicago, Maywood, IL 60153, USA; lbruegg@luc.edu (L.I.B.); mwang12@luc.edu (M.W.); akouta@luc.edu (A.K.); 2 Department of Cell and Molecular Physiology, Loyola University Chicago, Maywood, IL 60153, USA; lcribbs@luc.edu; 3 Department of Thoracic and Cardiovascular Surgery, Loyola University Chicago, Maywood, IL 60153, USA; JSCHWA1@lumc.edu

**Keywords:** smooth muscle, β-adrenergic receptor, Kv7 voltage-activated potassium channel, protein kinase A

## Abstract

β-adrenergic receptor (βAR) activation promotes relaxation of both vascular and airway smooth muscle cells (VSMCs and ASMCs, respectively), though the signaling mechanisms have not been fully elucidated. We previously found that the activity of Kv7.5 voltage-activated potassium channels in VSMCs is robustly enhanced by activation of βARs via a mechanism involving protein kinase A (PKA)-dependent phosphorylation. We also found that enhancement of Kv7 channel activity in ASMCs promotes airway relaxation. Here we provide evidence that Kv7.5 channels are natively expressed in primary cultures of human ASMCs and that they conduct currents which are robustly enhanced in response to activation of the βAR/cyclic adenosine monophosphate (cAMP)/PKA pathway. MIT Scansite software analysis of putative PKA phosphorylation sites on Kv7.5 identified 8 candidate serine or threonine residues. Each residue was individually mutated to an alanine to prevent its phosphorylation and then tested for responses to βAR activation or to stimuli that elevate cAMP levels. Only the mutation of serine 53 (S53A), located on the amino terminus of Kv7.5, significantly reduced the increase in Kv7.5 current in response to these stimuli. A phospho-mimic mutation (S53D) exhibited characteristics of βAR-activated Kv7.5. Serine-to-alanine mutations of 6 putative PKA phosphorylation sites on the Kv7.5 C-terminus, individually or in combination, did not significantly reduce the enhancement of the currents in response to forskolin treatment (to elevate cAMP levels). We conclude that phosphorylation of S53 on the amino terminus of Kv7.5 is essential for PKA-dependent enhancement of channel activity in response to βAR activation in vascular and airway smooth muscle cells.

## 1. Introduction

Voltage-activated Kv7 potassium channels, encoded by *KCNQ* genes, mediate “M-currents”, which have been implicated in the regulation of neuronal excitability by G protein-coupled receptor agonists [1,2] and more recently identified as intermediates in smooth muscle signal transduction [3,4]. The *KCNQ1*–*5* gene products (Kv7.1–Kv7.5 α-subunits) assemble as homo- or hetero-tetramers to form functional channels [5]. Each α-subunit has a cytosolic N-terminus, 6 transmembrane domains, and a cytosolic C-terminus. As modulators of cell excitability, Kv7 channels are tightly regulated. Suppression of Kv7 channel activity increases cell excitability, whereas augmentation of the Kv7 channel activity decreases excitability [6]. 

Smooth muscle cells mainly express *KCNQ1*, *KCNQ4*, and *KCNQ5* [7], though functional channels appear to be formed predominantly by the *KCNQ4* and *KCNQ5* gene products (Kv7.4 and Kv7.5 α-subunits), with no apparent contribution from *KCNQ1*/Kv7.1 [3]. We recently found that the activity of homomeric Kv7.5 but not homomeric Kv7.4 channels in VSMCs was strongly enhanced via the activation of the βAR/G_s_/cAMP/PKA pathway and this effect was associated with PKA-dependent phosphorylation of Kv7.5 channel α-subunits [8]. The PKA phosphorylation sites of Kv7.5 channels and mechanisms underlying the phosphorylation-induced increase in currents remain unknown.

The Kv7.5 α-subunit contains a reported consensus site for PKA phosphorylation at serine residue 600 (S600) on its C-terminal segment [9]. However, evaluation of amino acid sequences using MIT Scansite software [10] revealed 10 additional putative PKA phosphorylation sites in Kv7.5, only three of which have homologous residues in Kv7.4. Of the eight putative phosphorylation sites that are found in Kv7.5 but not in Kv7.4, two sites, T32 and S53, are located on the Kv7.5 N-terminus and six are on its C-terminus. 

In the present study we found that M-currents in primary cultured human airway smooth muscle cells (HASMCs) exhibit pharmacological and electrophysiological properties of Kv7.5 homomeric channels and can be robustly enhanced by therapeutic bronchodilators that activate the βAR/G_s_/cAMP/PKA pathway. We also provide evidence that N-terminal S53 is an essential site for PKA-dependent regulation of smooth muscle Kv7.5 channel α-subunits.

## 2. Results

### 2.1. Expression and Functional Characteristics of Kv7 Channels in Cultured HASMCs

**Figure 1:**
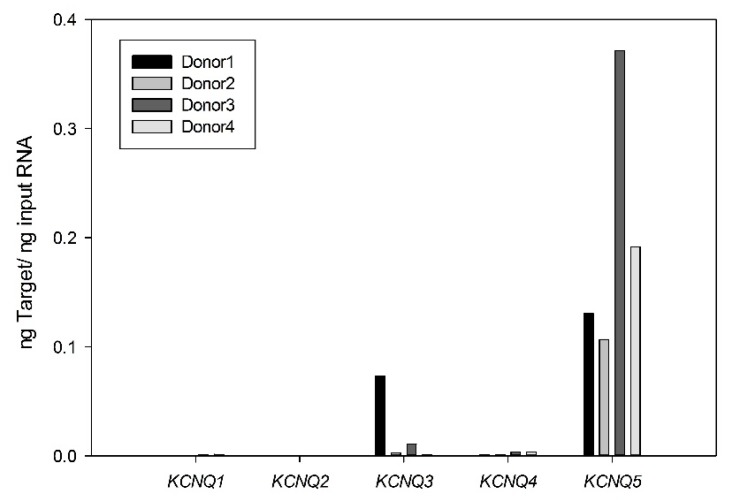
Expression of different *KCNQ* isoforms in cultured human airway smooth muscle cells (HASMCs). Expression levels of mRNAs for *KCNQ1*–*5* were estimated using quantitative real time RT-PCR in HASMCs. Target abundance values represent four different donor cultures (means from triplicate measurements from each sample).

We previously recorded the smooth muscle equivalent of M-currents in cultured HASMCs and attributed them to functional Kv7 channels based on the ability of the Kv7.2–7.5 activator retigabine, and Kv7 channel blockers (XE991 and linopirdine), to enhance and suppress, respectively, the non-inactivating voltage-dependent outwardly rectifying potassium currents [11]. To determine the molecular basis for these currents, we performed quantitative real time reverse transcriptase polymerase chain reaction (qRT-PCR) analysis, which revealed that cultured HASMCs expressed the five *KCNQ* genes in relative abundances of *KCNQ5* >> *KCNQ3* > *KCNQ4* > *KCNQ2* = *KCNQ1* (Figure 1).

**Figure 2:**
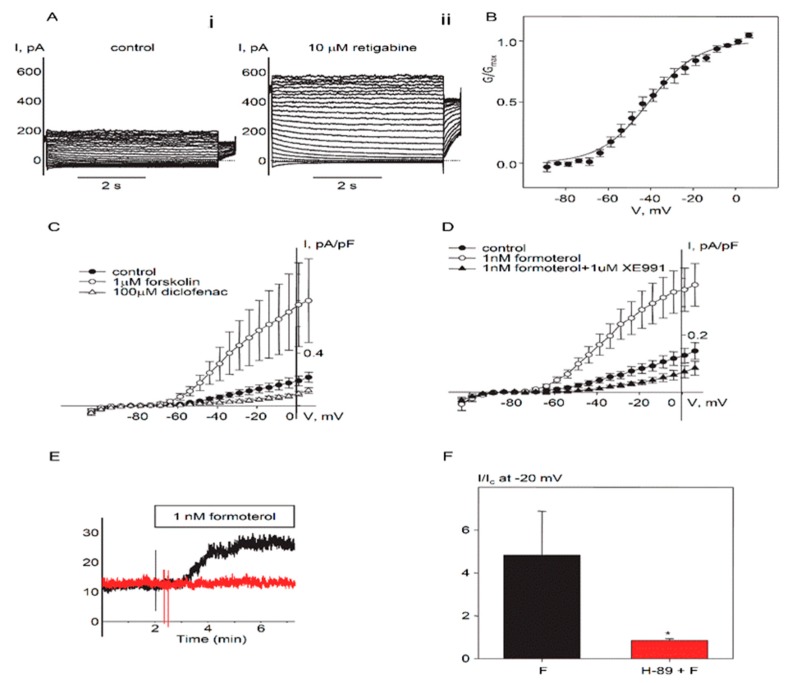
Protein kinase A (PKA)-dependent regulation of endogenous Kv7.5 currents in cultured HASMCs. (**A**) Representative current traces recorded in a single HASMC (Capacitance = 281 pF) before (i. control) and 5 min after addition of 10 µM retigabine (ii). (**B**) Mean fractional conductance plot calculated from steady-state endogenous Kv7 currents fitted to a Boltzmann distribution (V_0.5_ = −40.8 mV, *n* = 10). (**C**) I–V relationships of Kv7 currents recorded in HASMCs before (control, filled circles, *n* = 5), after 5 min treatment with 1 µM forskolin (open circles, *n* = 4), and after 5 min treatment with diclofenac (100 µM, open triangles, *n* = 4). (**D**) I–V relationships of Kv7 currents recorded in HASMCs before (control, filled circles, *n* = 7), after 5 min treatment with 1 nM formoterol (open circles, *n* = 7), and after 5 min treatment with Kv7 channel blocker XE991 (1 µM) in the presence of 1 nM formoterol (closed triangles, *n* = 3). (**E**) Representative time-courses of 1 nM formoterol application recorded at −20 mV in a single untreated HASMC (black, Capacitance = 39 pF) and a HASMC pretreated with 10 µM H-89 (red, C = 127 pF). (**F**) Relative formoterol-induced enhancement of the current recorded at −20 mV in untreated HASMCs (black bars, *n* = 7) and in HASMCs pretreated with H-89 (10 µM for 20 min, red bar, *n* = 4). * Significant difference from control (*p* < 0.01, Mann-Whitney Rank Sum Test).

Earlier research suggested that the endogenous Kv7 current in cultured HASMCs was suppressed by histamine in a PKC-dependent manner [11], which is a hallmark of smooth muscle Kv7.5 channels [11,12]. Additional characterization of the endogenous current revealed an absence of time-dependent inactivation, slow kinetics of deactivation and activation, sensitivity to the Kv7.2–Kv7.5 activator retigabine (Figure 2A), and relatively negative voltage dependence of activation (V_0.5_ = −40.8 ± 2.2 mV, *n* = 10, Figure 2B), further supporting a contribution of Kv7.5. To determine if endogenous Kv7 current in cultured HASMCs reflects predominantly Kv7.5 channel activity, we tested the effects of diclofenac, a drug previously found to distinguish among various Kv7 channel configurations [13]. Diclofenac (100 µM) robustly and rapidly inhibited the currents (Figure 2C), indicative of functional homomeric Kv7.5 channels [13]. We also measured the regulation of native HASMC Kv7 currents by the βAR/G_s_/cAMP/PKA pathway (also a characteristic feature of Kv7.5). Consistent with a major contribution of Kv7.5, application of forskolin (1 µM), a direct activator of adenylyl cyclase, robustly enhanced retigabine-sensitive currents (Figure 2C). 

### 2.2. Regulation of Endogenous Kv7.5 Currents in Cultured HASMCs by β-Adrenergic/Gs/cAMP/PKA Pathway

The βAR/G_s_/cAMP/PKA pathway is a therapeutic target for obstructive airway diseases. We tested the long-acting β-adrenergic agonist formoterol (1 nM), which is used in therapy for respiratory diseases such as asthma and chronic obstructive pulmonary disease and found that it also potentiated endogenous Kv7 current in HASMCs (Figure 2D). Pretreatment of HASMCs with PKA inhibitor H-89 (10 µM for 20 min) completely prevented enhancement of endogenous Kv7 current by formoterol (Figure 2E,F). 

### 2.3. Identification of PKA Phosphorylation Sites on Human Kv7.5 Channels

**Figure 3:**
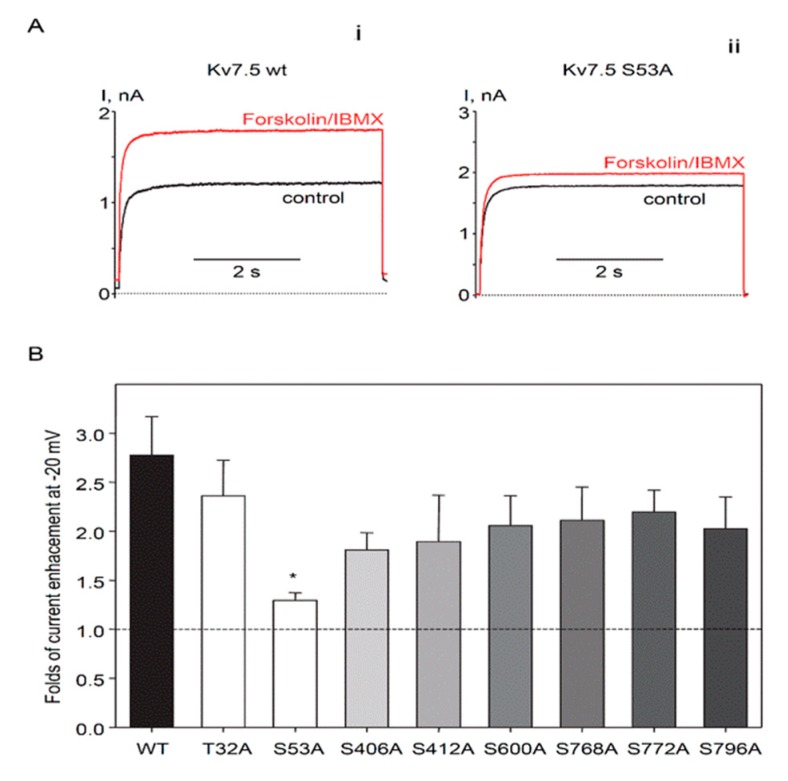
Putative PKA phosphorylation site Ser53, on the N-terminus of the Kv7.5 channels, is required for enhancement of Kv7.5 currents by forskolin/IBMX. (**A**) Representative traces of current through exogenous wild-type Kv7.5 channels (i) and through Kv7.5 S53A mutant channels (ii) recorded at −20 mV before (black, C = 14.2 pF) and after (red, C = 28.8 pF) application of forskolin (10 µM) in combination with IBMX (500 µM) for 5 min. (**B**) Relative forskolin/IBMX-induced enhancement of the current recorded at −20 mV for different phosphomutants in comparison to wild-type Kv7.5 channels (WT, black bar, *n* = 10), Kv7.5T32A (*n* = 5), Kv7.5S53A (*n* = 7), Kv7.5S406A (*n* = 6), Kv7.5S412A (*n* = 6), Kv7.5S600A (*n* = 6), Kv7.5S768A (*n* = 6), Kv7.5S772A (*n* = 6), Kv7.5S796A (*n* = 4). White bars represent N-terminal mutant, grey bars of different shades represent C-terminal mutants. * Significant difference from wild-type (*p* = 0.03, One Way ANOVA on Ranks).

To identify PKA phosphorylation sites involved in positive regulation of Kv7.5 channel α-subunits, we mutated all 8 distinct putative PKA phosphorylation sites individually, from serine or threonine to alanines: 2 sites located on the N-terminus (T32A and S53A) and 6 sites located on C-terminus (S406A, S412A, S600A, S768A, S772A, and S796A). Each Kv7.5 mutant construct produced functional channels when expressed in A7r5 vascular smooth muscle cells, based on detection of exogenous currents with the characteristics of Kv7.5 (negative threshold of activation and slow kinetics of activation/deactivation). Voltages of half activation for all mutants were negative to −46 mV, which tended to be more negative than V_0.5_ of wild-type Kv7.5 channels (−43.7 ± 2.2 mV, *n* = 10) though without reaching statistical significance. Currents through the mutant Kv7.5 channels were measured before and after treatment with a combination of forskolin (10 µM) and IBMX (non-selective phosphodiesterase inhibitor, 500 µM) to fully activate the cAMP/PKA pathway (Figure 3). Only mutation of Kv7.5 S53 significantly reduced the increase in current amplitude induced by forskolin/IBMX (29.6 ± 7.7% increase over baseline for S53A in comparison to 177 ± 39.5% increase of current amplitude through wild-type Kv7.5 channels, Figure 3A,B). 

**Figure 4:**
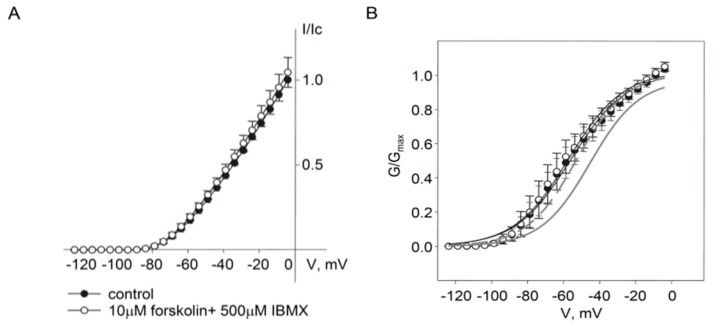
Phosphomimetic mutant Kv7.5S53D has negatively shifted voltage-dependence of activation and is insensitive to forskolin/IBMX. (**A**) I–V relationships of Kv7.5 S53D current recorded before (filled circles, *n* = 4) and after (open circles, *n* = 4) application of forskolin (10 µM) in combination with IBMX (500 µM) for 5 min. (**B**) Mean fractional conductance plots calculated from tail currents measured in control (filled circles) and in the presence of forskolin/IBMX (open circles), fitted to a Boltzmann distribution (V_0.5_ = −56.8 ± 5.3 mV in the absence and −58.4 ± 5.9 mV in the presence of forskolin/IBMX, *n* = 4); mean fractional conductance plots of wild-type Kv7.5 channels are shown in a gray solid line for control and gray dashed line in the presence of forskolin/IBMX.

We further tested whether mutation of S53 to aspartic acid (S53D) would mimic the effects of phosphorylation on Kv7.5. The Kv7.5 S53D mutant produced currents similar in density to wild-type currents but forskolin treatment did not induce an increase in current amplitude, consistent with an inability of PKA to phosphorylate the aspartic acid residue at the S53 site (Figure 4A). However, treatment with forskolin/IBMX also produced a negative shift of the activation curve for the wild-type channel (from −43.7 ± 2.2 mV to −53.3 ± 1.9 mV, *n* = 10) and this effect was mimicked by the S53D mutation. Forskolin/IBMX treatment had no further effect on the voltage-dependence of the S53D mutant (V_0.5_ = −56.8 ± 5.3 mV in the absence and −58.4 ± 5.9 mV in the presence of forskolin/IBMX, *n* = 4). 

**Figure 5:**
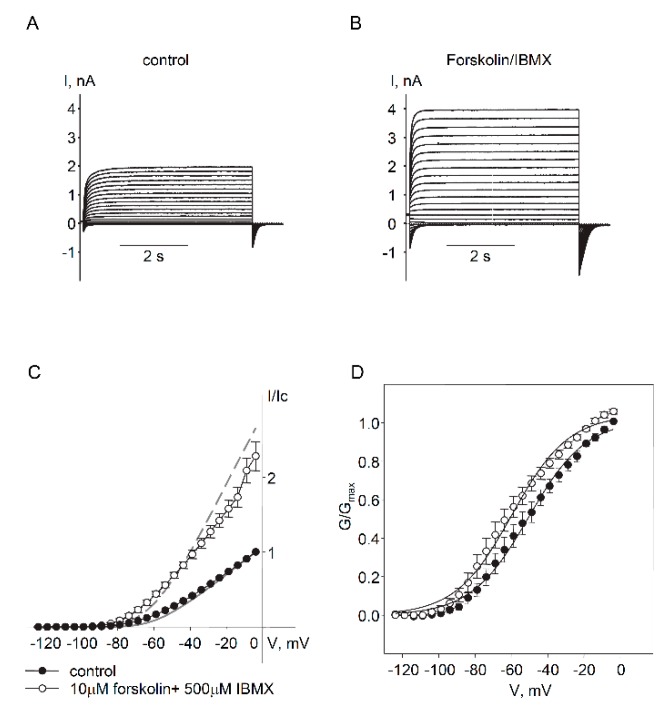
The enhancement of currents by forskolin/IBMX was not significantly affected by mutation of all six putative C-terminal PKA phosphorylation sites of Kv7.5 channels. Upper panels: representative current traces through mutant Kv7.5_C-term_6xS→A channel (C = 84 pF) in control (**A**) and in the presence of 10 µM forskolin in combination with 500 µM IBMX (**B**). (**C**) I–V relationships of Kv7.5_C-term_6xS→A current recorded before (filled circles, *n* = 5) and after (open circles, *n* = 5) application of forskolin (10 µM) in combination with IBMX (500 µM) for 5 min. I–V of wild-type Kv7.5 channels are shown in grey solid line (control) and grey dashed line (forskolin/IBMX-treated) for comparison. (**D**) Mean fractional conductance plots calculated from tail currents measured before (control, filled circles) and after 5 min in forskolin/IBMX (open circles), fitted to a Boltzmann distribution (V_0.5_ = −51.4 ± 3.2 mV in the absence, and −61.7 ± 3.2 mV in the presence of forskolin/IBMX, *n* = 5, *p* = 0.016, paired Student’s *t*-test).

We noticed diminished enhancement of currents through C-terminal S→A mutants in response to application of forskolin/IBMX that did not reach significance in each individual case. We created a combined mutant *KCNQ5* construct to encode a channel with all six of the putative C-terminal PKA phosphorylation sites mutated to alanines (Q5_C-term_6xS→A). The sextuple S→A mutant produced functional channels with properties similar to wild-type Kv7.5 (V_0.5_ = −51.4 ± 3.2 mV, *n* = 5; Figure 5A,C,D). The Q5_C-term_6xS→A currents were enhanced by forskolin/IBMX to approximately the same extent as the individual C-terminal S→A mutants, not significantly different from wild-type Kv7.5 (Figure 5).

## 3. Discussion

Kv7 channels in vascular and airway smooth muscle cells have been increasingly recognized as important regulators of their electrical excitability and as potential therapeutic targets for the treatment of diseases involving altered smooth muscle function [3]. The extent to which these channels may be regulated by activation of βARs and the mechanisms underlying such regulation are not well defined. Filling these gaps in our knowledge is of particular importance considering that the βAR/G_s_/cAMP/PKA pathway has been established as an important therapeutic target, particularly for treatment of obstructive airway diseases. The present study reveals that Kv7.5 channels are prominent in HASMCs and provides new evidence that the βAR/G_s_/cAMP/PKA pathway targets these channels via phosphorylation of a specific serine residue located on the amino terminus of the channel’s α-subunits.

Considering that multiple *KCNQ* genes may be expressed in HASMCs, it was important to determine which of these gene products contributes to the Kv7 currents measured in these cells. *KCNQ5* message levels were found to be the most abundant and the pharmacological and electrophysiological characteristics of the native Kv7 currents were consistent with a predominant functional contribution of Kv7.5 α-subunits. Stimulation of the βAR/G_s_/cAMP/PKA pathway increased current amplitude, an effect which was previously observed for cloned Kv7.5 but not Kv7.4 [8]. Diclofenac induced a rapid and nearly complete suppression of the currents, which is also characteristic for cloned Kv7.5 [13]. Both diclofenac and its close structural analogue meclofenamate were reported to modestly enhance Kv7.3 currents in the Chinese hamster ovary (CHO) cell expression system [14]. Thus, although *KCNQ3* mRNA was detectably expressed in some HASMC samples, Kv7.3 channels are unlikely to contribute appreciably to the diclofenac-inhibited whole cell currents recorded in the HASMCs. A major Kv7.4 contribution appears unlikely considering that *KCNQ4* was negligibly expressed, currents through Kv7.4 channels expressed in smooth muscle cells were previously found to be insensitive to the cAMP/PKA pathway and Kv7.4 currents were robustly enhanced by treatment with diclofenac [8,13]. Although heteromeric Kv7.4/Kv7.5 channels have been described in arterial smooth muscle cells [15,16], available evidence suggests that currents through Kv7.4/Kv7.5 channels expressed in A7r5 cells are only modestly enhanced by forskolin/IBMX and partially inhibited by diclofenac treatments [8,13]. The voltage-dependence for activation varies among Kv7 channel subtypes when expressed in mammalian cells, with Kv7.5 activating at more negative voltages than other subtypes. The V_0.5_ of activation of HASMC Kv7 currents (−41 mV) is most similar to that reported for cloned Kv7.5 channels expressed in mammalian cells (−44.2 ± 1.7 mV; −46 ± 3 mV [13,17]) and considerably more negative than that of Kv7.4 (−30.6 ± 1.2 mV [13]), or Kv7.3 (−37.3 ± 1.3 mV [18]). All told, the experimental evidence suggests that the endogenous Kv7 currents measured in HASMCs are elicited predominantly via Kv7.5 channels.

The expression pattern for *KCNQ* genes in HASMCs differed considerably from a previous study that had measured *KCNQ* expression in extracts prepared from strips of human trachealis muscle [19]. In that earlier study, the expression pattern was noted to be highly variable but on average *KCNQ1* was most abundant, *KCNQ4* and *KCNQ5* were expressed at approximately equal levels, and there was little or no detectable expression of *KCNQ2* or *KCNQ3*. The differences in expression between muscle strips and cultured cells could relate to phenotypic changes associated with cell culture or to the presence of non-smooth muscle cell types in different proportions between cultured cells and muscle strips. As noted previously, the Kv7 currents recorded from freshly dissociated human tracheal myocytes, like those from cultured HASMCs, were enhanced by retigabine, a drug that enhances currents from Kv7.2–Kv7.5 subtypes, but not Kv7.1 channels. Thus, the endogenous Kv7 currents cannot reasonably be attributed to Kv7.1 channels in either case.

Whether the currents measured in the cultured HASMCs are representative of functional channels that regulate airway smooth muscle contractility in the bronchioles of a human lung is more difficult to discern. There is now abundant evidence that pharmacological activators of Kv7.2–Kv7.5 (but not Kv7.1) are very effective dilators of bronchioles in precision-cut lung slices from human, guinea pig, rat, and mouse lungs [19,20,21]; and a pharmacological blocker of Kv7 channels was found to robustly constrict human bronchioles [19]. The actions of G_q/11_-coupled receptor agonists to suppress native Kv7 currents in human, guinea pig, and mouse airway myocytes [11,19,20,21] are consistent with their PKC-dependent inhibitory effects on cloned Kv7.5 channels [11] (but not cloned Kv7.4 channels [15]) and their well-known ability to constrict human bronchioles [22]. The same agonists (histamine and methacholine), were found to induce PKC-dependent phosphorylation of Kv7.5 protein in human trachealis or bronchus muscle strips [11]. The results presented here further suggest that the ability of G_s_-coupled receptor agonists to enhance native HASMC Kv7 currents is convergent with their PKA-dependent enhancement of cloned Kv7.5 currents and their well-known bronchorelaxant effects in human lungs [22]. 

Previous research on VSMCs and ASMCs has revealed numerous signaling mechanisms that can potentially contribute to the stimulation of smooth muscle relaxation in response to both physiological and pharmacological βAR agonists. The contractile mechanism, i.e., actin-myosin cross-bridge cycling, is triggered by an increase in cytosolic Ca^2+^ concentration ([Ca^2+^]_cyt_); hence, mechanisms that reduce [Ca^2+^]_cyt_ will tend to relax smooth muscle. One such mechanism, supported by results described above, is an increase in outward potassium current via Kv7.5 channels, which would result in membrane hyperpolarization and consequently reduce influx of Ca^2+^ via L-type voltage-sensitive Ca^2+^ channels [23]. Previously, phosphorylation of Kv7.5 channel subunits by PKA was implicated in the enhancement of Kv7.5 currents by activation of the βAR/G_s_/cAMP/PKA pathway [8]. However, several additional mechanisms have been proposed, including PKA-dependent phosphorylation of other substrates as well as cAMP-independent regulation of ion channels or other components of the Ca^2+^ signaling pathways; the end result is prevention or reduction of Ca^2+^ influx and/or release of intracellular Ca^2+^ stores, and ultimately reduced contraction (reviewed in [22,24]). There is also evidence that activation of βARs can lead to reductions in the actin-myosin cross-bridge cycling efficiency or its sensitivity to Ca^2+^, as a means to relax smooth muscle cells independently of changes in [Ca^2+^]_cyt_ [25]. The relaxation of bronchiolar smooth muscle in response to βAR agonists likely reflects contributions from many or all of these mechanisms. The efficacy of multiple structurally distinct pharmacological Kv7 channel activators as enhancers of native ASMC Kv7 currents and relaxers of bronchioles in precision-cut lung slices [20] suggests that an increase in Kv7 current is sufficient to induce bronchiolar relaxation. We therefore suppose that the effect of βAR agonists to induce a similar enhancement of Kv7 currents is an important contributor to the overall bronchiolar relaxation response to these agonists. However, a future challenge will be to determine exactly how much the Kv7 channel effects contribute to the full spectrum of actions triggered by βAR or other G_s_-coupled receptor agonists in healthy and diseased human bronchiolar smooth muscle.

We have provided evidence that Kv7.5 is the predominant Kv7 channel subunit in HASMCs and that the enhancement of Kv7.5 currents in response to stimulation of the βAR/G_s_/cAMP/PKA pathway is mediated by phosphorylation of S53 on the amino-terminal region of the Kv7.5 α-subunits. Mutation of S53 to either alanine (A) or aspartic acid (D) prevented forskolin-induced enhancement of the current. The S53A construct is expected to be non-productive in terms of PKA-dependent responses because it cannot be phosphorylated at that site (alanine is missing the required hydroxyl moiety) and alanine does not confer the negative charge that would result from phosphorylation of a serine at that site. In contrast, substitution at the serine site with a negatively charged aspartic acid residue (e.g., S53D) might be expected to serve as a phospho-mimic, as has been observed for other ion channels [26,27]. Although the absence of a forskolin response in the case of S53D is expected because the site is not available for PKA-mediated phosphorylation, the anticipated phospho-mimic effect would be that the currents are already enhanced-mimicking S53 phosphorylation even in the absence of stimulation of the βAR/G_s_/cAMP/PKA pathway. This would be apparent as an increased amplitude of the currents but the variability of current densities in our overexpression system precludes any conclusion in this regard. A negative shift in the voltage dependence of activation of the S53D mutant relative to wild-type Kv7.5, which is not further affected by forskolin/IBMX treatment, is consistent with a phospho-mimic effect of the aspartate residue. In our previous study, we used isoproterenol to stimulate the βAR/G_s_/cAMP/PKA pathway and found that enhancement of Kv7.5 current was not accompanied by a negative shift of the activation curve, whereas in the present study we detected a significant 9.6 mV negative shift of activation of Kv7.5 channels in the presence of forskolin/IBMX. The discrepancy with our previous finding may be explained by more robust activation of the βAR/G_s_/cAMP/PKA pathway by forskolin/IBMX in comparison to isoproterenol [8]. 

Ten additional potential PKA phosphorylation sites on Kv7.5 were identified by the MIT Scansite software [10], though 3 of these were conserved in Kv7.4, which is not apparently regulated by the PKA pathway [8] and mutation of the remaining sites to alanines did not significantly reduce the PKA-dependent enhancement of Kv7.5 currents. Although we cannot rule out the possibility that additional phosphorylation sites, not identified by the Scansite software, play some role in the PKA-dependent regulation of Kv7.5, if we focus on S53 as a primary site of regulation, an important question would be: how does phosphorylation of this site confer an increase in Kv7.5 current? 

An increase in whole cell current amplitude at a given voltage is normally attributed to: (1) an increased probability that the channels are in the open state, (2) an increase in the conductances of the individual channels, and/or (3) an increase in the number of active channels at the plasma membrane. One physiological mechanism proposed for positively regulating the activity of Kv7 channels is by increasing their association with the membrane phospholipid phosphatidylinositol 4,5-bisphosphate (PIP_2_) [28]. PIP_2_ stabilizes the open state of Kv7 channels [29]. PKA-dependent phosphorylation of two serine residues (S27 and S92) on the N-terminal segment of the Kv7.1 channel was suggested to increase the affinity of the channel to PIP_2_ [30]. Activity of human Kv7.2 channels may also be enhanced by PKA-dependent phosphorylation at S52 of its N-terminus, as validated in an expression system [31]. However, there is little evidence that PKA-dependent phosphorylation can increase the affinity of the channels for PIP_2_. Another previous study of Kv7.2 identified two PKA phosphorylation sites on its C-terminal segment (S438 and S455) but phosphorylation at these sites was associated with a decrease in the apparent affinity for PIP_2_ [32]. Such an effect on PIP_2_ affinity would be expected to decrease the open probability of Kv7.2 channels. Stott et al. reported that isoproterenol increased the open probability of endogenous Kv7 channels in rat renal artery smooth muscle cells, based on single-channel activity recorded in the cell-attached configuration; single channel conductance was not obviously affected [33]. Whole cell currents recorded from HEK293 cells overexpressing Kv7.4 were also modestly enhanced by 250 µM 8-bromo-cAMP in the same study, though the effects of both isoproterenol and 8-bromo-cAMP were inhibited by gallein, a putative blocker of G protein βγ-subunit actions [33] (see additional discussion below). A βAR agonist-induced increase in the number of active channels present in the membrane was not apparent in the cell-attached recordings of Stott et al. [33], though it cannot be ruled out in our whole cell recordings from HASMCs.

Another proposed mechanism(s) whereby βAR activation may lead to enhancement of smooth muscle Kv7 currents involves direct interactions of G protein βγ-subunits with the Kv7 channel α-subunits [33]. The available evidence for Kv7.4 channels either natively expressed in renal artery myocytes or exogenously expressed in HEK293 cells suggests that the G protein βγ-subunits are constitutively associated with the Kv7.4 channels and are somehow mobilized by βAR activation to exert a stimulatory effect on the probability of Kv7.4 channel opening [33]. A role for PIP_2_ binding in this process has also recently been suggested [34]. It is not known whether the G protein βγ-subunit signaling mechanism is unique to Kv7.4 or whether it functions independently of the βAR/G_s_/cAMP/PKA signaling events. 

In summary, previous research has established that stimulation of βARs promotes relaxation of both VSMCs and ASMCs, though the signaling mechanisms involved are not fully elucidated. Activation of Kv7 potassium channels has also been found to be sufficient to induce smooth muscle relaxation. The present study provides evidence for a mechanistic link between these processes: βAR receptor activation results in PKA-dependent phosphorylation of S53 on the N-terminus of Kv7.5 channel subunits, which increases channel activity, and likely contributes to βAR agonist-induced relaxation of vascular and airway smooth muscle. Although many drugs are available to directly activate Kv7 channels and some of these are in clinical use, they generally have effects across a broad spectrum of tissues including the nervous system, limiting their utility for treatment of smooth muscle disorders. Understanding the physiological mechanisms by which smooth muscle Kv7 channel activity is regulated may lead to alternative strategies for modulating this activity specifically in vascular or airway smooth muscle and thereby to development of more effective therapies for vascular and airway diseases.

## 4. Materials and Methods

### 4.1. Expression Constructs

The adenoviral vector to express human Kv7.5 (Adv-hKCNQ5-FLAG) was created previously using the AdEasy Adenoviral Vector System (Stratagene, Jolla La, CA USA) [13]. Mutagenesis of the human Kv7.5 (in pIRES2-EGFP cDNA) was performed using QuikChange Site-Directed Mutagenesis Kit (Stratagene). Mutations (individual serine or threonine to alanine or aspartic acid) of Kv7.5 (S406A, S412A, S600A, S768A, S772A, S796A, T32A, S53A, S53D), and sextuple C-terminal S to A mutations (S406A-S412A-S600A-S768A-S772A-S796A) were confirmed by DNA sequencing. 

### 4.2. Cell Culture

Airway smooth muscle cells from human tracheas (HASMCs) were isolated and cultured as described previously [11], under guidelines established by the Loyola University Chicago Institutional Review Board. HASMCs were subcultured on 35–mm dishes and serum deprived for 24–72 h before use. A7r5 cells were cultured as described previously [8]. For overexpression studies, A7r5 cells subcultured to 70% confluence were infected with Adv-hKCNQ5-FLAG at a multiplicity of infection of 100 and used for electrophysiological experiments 3–14 days after infection. Mutant Kv7.5 constructs were introduced into subcultured A7r5 cells by transient transfection with Lipofectamine 3000^®^ transfection reagent according to the manufacturer’s protocol. A7r5 cells expressing mutant Kv7.5 channels were used for electrophysiological studies 3–14 days after transfection. A7r5 cells were selected for electrophysiological experiments based on the detection of EGFP fluorescence introduced by the expression vector. 

### 4.3. Quantitative Real Time Reverse-Transcriptase Polymerase Chain Reaction (qRT-PCR)

Total RNA was extracted from primary HASMC cell lines (four different donors) using RNeasy Plus Mini Kit (Qiagen, Germantown, MD, USA). Following reverse transcription using iScript™ Reverse Transcription Supermix (Bio-Rad, Hercules, CA, USA), the cDNAs were amplified and analyzed in triplicate using PowerUp SYBR Green Master Mix (Applied Biosystems, Foster City, CA, USA). Primer sequences for human *KCNQ1*–*KCNQ5* are listed in Table 1. Expression was quantified in 25 µL reactions consisting of: 12.5 µL PowerUp SYBR Green Master Mix (Applied Biosystems, Foster City, CA, USA), cDNA derived from 50 ng mRNA and 5 pmol each primer. Cycle parameters were typically 95 °C for 15 s, followed by 60 °C for 30 s (40 cycles), followed by a dissociation step to confirm a single PCR product. Standard curves were plotted using 10-fold serial dilutions of known amounts of plasmid DNA for each target. Primer efficiencies were determined from the slope of the standard curve dilution series for each of the *KCNQ* targets, using the formula:

Efficiency = −1 + 10 (−1/slope)

The correlation coefficients for all standard curves were >0.99, and slope values gave efficiencies ≥99% in all cases. The amount of target in a sample was estimated by extrapolating the triplicate average PCR threshold cycle to target values on the standard curve.

### 4.4. Patch-Clamp

Electrophysiological recordings in perforated patch voltage-clamp configuration were conducted as described previously [8,11]. Cells were plated on 25 mm round glass coverslips 5–15 min prior to the experiment. Experiments were performed with continuous perfusion of bath solution at room temperature. Command voltages were generated and currents recorded using an Axopatch 200B amplifier under control of PCLAMP10 software (Molecular Devices, LLC, San Jose, CA, USA). Series resistances were compensated for overexpressed channels. 

Voltage-dependence of wild-type and mutant Kv7.5 channel activation was estimated as described previously [8] from the instantaneous tail current amplitude, converted to conductance according to the equation: *G* = *I_tail_*/(−120 −*E_K_*), where *I_tail_* is the instantaneous tail current amplitude, −120 mV is the tail current step potential and *E_K_* is the reversal potential for potassium (−86 mV). Conductance plots in the absence (control) and in the presence of forskolin (10 µM) and IBMX (500 µM) for each experiment were fitted to a Boltzmann distribution: *G*(*V*) *= G_max_*/[1 + *exp*(*V*_0.5_ – *V)*/*k*], where *G* is conductance, *G_max_* is a maximal conductance, *V*_0.5_ is the voltage of half-maximal activation and *k* is the slope factor. Each conductance curve for each experiment was normalized by *G_max_* from the Boltzmann fit and *G*/*G_max_* curves from all corresponding individual experiments were averaged to produce conductance plots presented in the figures and fitted to the Boltzmann distribution without any constraints. Voltage dependence of endogenous current activation in HASMCs was determined by converting steady-state end pulse current to conductance, according to the equation: *G* = *I*/(*V* − *E_K_*), where *I* is the current amplitude, *V* is the step potential and *E_K_* is the reversal potential for potassium (−101 mV). 

### 4.5. Statistics

Data were analyzed using SigmaPlot (Systat Software, Inc., San Jose, CA USA) and are expressed as mean ± standard error of the mean (S.E.). Parameters measured before and after treatments were compared using a paired Student’s *t*-test. For comparisons between two independent groups, *t*-test or the *t*-test followed by the Mann-Whitney rank-sum test was used. Differences among multiple treatment groups were assessed by analysis of variance (ANOVA) followed by a Holm-Sidak post hoc test or ANOVA on ranks followed by multiple comparisons vs. control group (Dunn method). Differences were considered statistically significant with *p* values ≤ 0.05.

### 4.6. Material

Cell culture media were from MediaTech (Herndon, VA, USA). Fetal bovine serum was from Atlanta Biologicals. Antibiotic/antimycotic solution, Formoterol fumarate, 3-Isobutyl-1-methylxanthine (IBMX) were from Sigma-Aldrich. Amphotericin B, H-89 dihydrochloride were from Calbiochem. Forskolin was from Tocris Bioscience (Minneapolis, MN, USA). Retigabine was from Alomone Labs (Jerusalem, Israel). The AdEasy Adenoviral Vector System was from Stratagene. The QuikChange Site-Directed Mutagenesis Kit was from Agilent Technologies (Santa Clara, CA, USA). The human *KCNQ5* cDNA (accession number: AF202977, originally in the insect cell expression vector pMT) was a generous gift from Dr. T. Jentsch at the Max-Delbrück-Centrum for Molecular Medicine (Berlin, Germany).

## Figures and Tables

**Table 1:** Human qRT-PCR Primers.hyphen.

Gene	Primer Sequence	Product Size (bp)
*KCNQ1*	F: 5′-AAC CTC ATG GTG CGC ATC AAG-3′ R: CCG CGA TCC TTG CTC TTT TCT G -3′	101
*KCNQ2*	F: 5′-CGG AAA CCG TTC TGT GTG ATT GAC-3′ R:5′-ATC C GCA GAA TCT GCA GGA AG C-3′	131
*KCNQ3*	F: 5′-CCA CGC CAA AAC ACA AGA AGT CT-3′ R: 5′-TGA TGT GGA TGG TCT GGC TAC A-3′	101
*KCNQ4*	F: 5′-TGCG ACC GTA CGA CGT GAA G-3′ R: 5′-CAA TTT GGT CCA CCC GAG TTT GC-3′	102
*KCNQ5*	F: 5′-CCATCCCTGAGCACACAAAATTGGC -3′ R: CACCCTGACACATAAACCCTG-3′	109

F = Forward; R = Reverse.

## Abbreviations

ANOVA	analysis of variance
ASMC	Airway smooth muscle cell
cAMP	Cyclic adenosine monophosphate
HASMCs	Human airway smooth muscle cells
IBMX	3-Isobutyl-1-methylxanthine
PIP_2_	Phosphatidylinositol 4,5-bisphosphate
PKA	Protein kinase A
PKC	Protein kinase C
qRT-PCR	Quantitative real time reverse-transcriptase polymerase chain reaction
S.E.	Standard error of the mean
VSMC	Vascular smooth muscle cell
βAR	β-adrenergic receptor
